# Organoids as Model Systems to Investigate Circadian Clock-Related Diseases and Treatments

**DOI:** 10.3389/fgene.2022.874288

**Published:** 2022-04-26

**Authors:** Suengwon Lee, Christian I. Hong

**Affiliations:** Department of Pharmacology and Systems Physiology, University of Cincinnati College of Medicine, Cincinnati, OH, United States

**Keywords:** circadian rhythms, organoids, circadian medicine, chronotherapy, circadian drug screening

## Abstract

Circadian rhythms exist in most cell types in mammals regulating temporal organization of numerous cellular and physiological processes ranging from cell cycle to metabolism. The master clock, suprachiasmatic nucleus (SCN) in the hypothalamus, processes light input and coordinates peripheral clocks optimizing organisms’ survival and functions aligning with external conditions. Intriguingly, it was demonstrated that circadian rhythms in the mouse liver can be decoupled from the master clock under time-restricted feeding regimen when food was provided during their inactive phase. Furthermore, mouse liver showed clock-controlled gene expression even in the absence of the master clock demonstrating independent functions of peripheral clocks apart from the SCN. These findings suggest a dynamic relationship between the master and peripheral clocks and highlight potential functions of peripheral clocks independent of the master clock. Importantly, disruption of circadian rhythms correlates with numerous human ailments including cancer and metabolic diseases, suggesting that diseases may be exacerbated by disruption of circadian rhythms in the SCN and/or peripheral clocks. However, molecular mechanisms providing causative links between circadian rhythms and human diseases remain largely unknown. Recent technical advances highlighted PCS- and tissue-derived 3-dimensional organoids as *in vitro* organs that possess numerous applications ranging from disease modeling to drug screening. In this mini-review, we highlight recent findings on the importance and contributions of peripheral clocks and potential uses of 3D organoids investigating complex circadian clock-related diseases.

## Introduction

Circadian rhythms are periodic events that occur with a period of approximately 24 h regulating temporal organizations of diverse cellular and physiological processes such as metabolism, immune response, and sleep/wake cycles. In the past four decades, extensive research uncovered detailed molecular mechanisms of circadian rhythms in several eukaryotic model organisms including *Neurospora crassa, Arabidopsis Thaliana*, *Drosophila melanogaster*, and *Mus musculus* (Dunlap and Loros, 2017; Cox and Takahashi, 2019; Creux and Harmer, 2019; Patke et al., 2020), and demonstrated that eukaryotic systems possess conserved molecular blueprint that consist of time-delayed transcriptional-translational feedback loop (TTFL) (Hurley et al., 2016), which generates autonomous circadian oscillations even at single cell level (Nagoshi et al., 2004). The endogenous circadian clocks enable organisms to process external time cues such as light/dark cycles, and coordinate temporal organization of physiological processes anticipating recurring daily events. Comparative analyses of circadian clock mechanisms in different organisms have been previously reviewed (Hurley et al., 2016; Saini et al., 2019).

Briefly, molecular mechanisms of mammalian circadian machinery involve heterodimeric basic helix-loop-helix (bHLH) PER-ARNT-SIM (PAS) domain containing transcription factors, CLOCK (circadian locomotor output cycles kaput) and BMAL1 (brain and muscle Arnt-like protein-1), which serve as positive elements activating negative elements, *Period* (*Per1, Per2, Per3*) and *Cryptochrome* (*Cry1, Cry2*) genes. CLOCK/BMAL1 binds to E-box motifs on target gene promoters including other transcription factors, which regulate a large number of clock-controlled genes (CCGs) (Takahashi, 2017). PER and CRY proteins form complexes, and translocate into the nucleus where they inhibit CLOCK/BMAL1 forming a time-delayed negative feedback loop. PER and CRY undergo progressive phosphorylations and subsequent ubiquitination and degradation (Reischl et al., 2007; Siepka et al., 2007; Yoo et al., 2013), which relieves the repression on CLOCK/BMAL1. Nuclear receptors, RORs (RORα, RORβ, RORγ) and REV-ERBs (REV-ERBα, REV-ERBβ), form additional feedback loops with BMAL1 regulating circadian rhythms and metabolism (Preitner et al., 2002; Cho et al., 2012). Molecular mechanisms of mammalian circadian rhythms have been extensively reviewed previously (Partch et al., 2014; Cox and Takahashi, 2019; Rosensweig and Green, 2020).

In mammals, the suprachiasmatic nucleus (SCN) within the hypothalamus serves as the master clock coordinating peripheral clocks in different parts of the body (Ralph et al., 1990). The SCN contains approximately 20,000 neurons that generate synchronized oscillations *via* intercellular signaling and orchestrate rhythmic physiological activities. Light is one of the strongest external time cues that travels through rods, cones, and intrinsically photosensitive retinal ganglion cells (ipRGCs) to the SCN *via* retinal innervation inducing phase shift and entrainment of circadian rhythms (Lazzerini Ospri et al., 2017). Either genetic or physical ablation of circadian rhythms in the SCN abolishes rhythmic behavioral activities and disrupts tissue-specific clock functions in peripheral organs (Ralph et al., 1990). Intriguingly, mice that are forced to eat only during their inactive phase results in food-induced phase resetting of circadian rhythms in peripheral organs (e.g., liver, kidney, heart, etc.) decoupling the phase relationship between the master and peripheral clocks (Damiola et al., 2000). Current understanding of the SCN such as intercellular coupling, light input signaling pathways, and hierarchical regulation of peripheral clocks by the SCN have been extensively reviewed elsewhere (Lazzerini Ospri et al., 2017; Hastings et al., 2019). In this mini-review, we focus on the importance of peripheral clocks and potential uses of 3-dimensional organoids addressing roles of circadian rhythms in complex human diseases.

## Peripheral Clocks

Genome-wide analysis of mouse and human gene expression profiles demonstrated peripheral organ-specific CCGs uncovering organ-specific functions of circadian rhythms. Specifically, the transcriptome of 12 mouse organs showed that up to approximately 40% of protein coding genes are CCGs (Zhang et al., 2014). And more recently, gene expression analysis of 13 human tissues using the cyclic ordering by periodic structure (CYCLOPS) algorithm uncovered tissue-specific regulation of CCGs including numerous drug target genes, which highlighted the importance of circadian rhythms in drug treatment and response (Ruben et al., 2018). Tissue-specific regulations of CCGs involve interactions of circadian clock transcription factors with tissue-specific transcription factors (Meireles-Filho et al., 2014) and tissue-specific chromatin loops enabling tissue-specific rhythmic gene expression profiles (Yeung et al., 2018). It is important to note that the above data is a result of an integrated output from both systemic and peripheral clocks. Peripheral organs receive temporal cues from the SCN *via* humoral and neuronal signals, which enables them to align and coordinate tissue-specific functions with the master clock.

Surprisingly, it was discovered that peripheral tissues can directly process external time cues such as light, temperature, and nutrient information regulating entrainment of tissue-specific circadian rhythms and CCGs. Specifically, temperature cycles entrain circadian rhythms in the liver and cultured fibroblasts *via* heat-shock factor 1 (HSF1) (Brown et al., 2002; Saini et al., 2012). Low-amplitude endogenous body temperature cycles were sufficient to induce temperature-dependent alternative splicing that could potentially regulate a large number of gene expression in the mouse liver (Preussner et al., 2017). Intriguingly, transcriptome profiles in the epidermis and the liver show CCGs in the absence of circadian rhythms in other tissues including the SCN under light-dark cycle (Koronowski et al., 2019; Welz et al., 2019), which suggest light entrainment of peripheral clocks independent of the SCN. Furthermore, the mouse liver clock maintains synchronized oscillations in the SCN-lesioned mice and “hepatocyte clock-only mice” even in the absence of external time cues, which indicates that peripheral clocks could function independent of the SCN (Sinturel et al., 2021). In addition, neuropsin (OPN5) and encephalopsin (OPN3) regulate entrainment of murine skin (Buhr et al., 2019) and light sensing of adipocytes (Nayak et al., 2020) providing evidence of direct photosensing pathways in peripheral systems.

Nutrient is another strong external cue that could alter the period and phase of canonical clock genes and the composition of CCGs in peripheral organs. High-fat diet (HFD) induces diet-induced obesity and disrupts circadian rhythms in mice. HFD lengthens the free running period of circadian rhythms, reduces amplitude of canonical clock genes in fat and liver, and changes diurnal profiles of key metabolic genes in hypothalamus, fat, and liver (Kohsaka et al., 2007). Furthermore, HFD induces reorganization of CCGs in the mouse liver resulting in both loss and gain of oscillating transcripts compared to normal chow diet indicating that the composition of CCGs is dynamic (Eckel-Mahan et al., 2013). Intriguingly, mice that are fed HFD for 8 h during their active phase, time-restricted feeding (TRF), did not experience obesity nor other metabolic conditions that are commonly observed in mice that are fed HFD ad libitum (Hatori et al., 2012). It is important to note that TRF entrained the liver clock and metabolic components to provide protection against HFD highlighting the importance of peripheral clock function (Hatori et al., 2012). The above supporting data underscores the importance of peripheral clocks, dynamic relationship between the master and peripheral clocks, and potential impact of disrupted peripheral clocks in human diseases.

Aforementioned mouse models that enable tissue-specific rescue of circadian rhythms will be instrumental to uncover peripheral organ-specific functions of circadian rhythms. However, it is still difficult to determine functions of peripheral clocks in human subjects and how those functions may change as human diseases emerge with conventional experimental models. Recent advancements of patient-derived 3-dimensional organoids indicate that human organoid models will play a critical role determining functions of peripheral clocks, remodeling of clock-controlled processes in different diseases, and circadian target genes for potential chronotherapy.

## Organoids to Investigate Clock-Related Complex Human Diseases

Previous findings indicated correlation between cancer and expression of core clock genes in breast, prostate, pancreatic, liver, colorectal, and lung cancer. For example, reduced expression of *Period* (i.e., *Per1*, *Per2*, and/or *Per3*) genes have been observed in sporadic breast tumors (Winter et al., 2007), hepatocellular carcinoma tissues (Lin et al., 2008), and colon cancer cells of primary colorectal tumors (Mostafaie et al., 2009). On the other hand, overexpression of *Per2* and downregulation of *Bmal1* has been shown to inhibit growth of human pancreatic cancer cells and glioblastoma stem cells, respectively (Oda et al., 2009). Furthermore, human cancers show altered circadian rhythms (Shilts et al., 2018), but consequences of human diseases impacting the core circadian machinery and clock-controlled signaling pathways remain largely unknown.

One of the limitations of investigating human disease-specific mechanisms of circadian rhythms is the lack of human model systems that enable diseased tissue-specific investigations of altered circadian rhythms and clock-controlled signaling pathways. Transformed homogenous 2D human cell lines such as HeLa and U2OS provide cost effective access to human cells but do not possess multicellular characteristics of *in vivo* organs and cannot model/represent disease-specific alterations. Furthermore, previous studies using NIH3T3 and U2OS cells demonstrated that they possess less than a dozen rhythmic genes (Hughes et al., 2009), which suggested that transformed cell lines are not ideal systems investigating disruption of clock-controlled signaling pathways. Recently, human gene expression data from the National Center for Biotechnology Information Gene Expression Omnibus (GEO) have been used to extract rhythmic signatures using cyclic ordering by periodic structure (CYCLOPS) algorithm (Anafi et al., 2017). However, CYCLOPS requires a minimum of 250 samples to identify rhythmic genes, and still requires human *in vitro* systems to test potential chronotherapeutic regimens for applications of translational circadian medicine. In this regard, patient tissue-derived organoids present as attractive models that enable disease-specific interrogation of remodeling of clock-controlled genes and signaling pathways and *in vitro* validations of chronotherapeutic regimens.

Organoids are *in vitro* multicellular culture systems mimicking cellular and physiological functions of specific organs depending on the source and development of those 3D cultures. Organoids can be derived from either pluripotent stem cells (PSCs) or adult stem cells (ASCs). Human PSCs can be used to derive different types of human organoids including nerve/sensory (brain, optic/retina) (Meyer et al., 2011; Lancaster et al., 2013), endocrine/gland (thyroid, mammary gland, prostate, fallopian tube, endometrium) (Ma et al., 2015; Qu et al., 2017; Yucer et al., 2017; Hepburn et al., 2020; Cheung et al., 2021), respiratory/cardiovascular (lung, heart, blood vessel) (Dye et al., 2015; Wimmer et al., 2019; Drakhlis et al., 2021), and gastrointestinal tract/digestive (stomach, liver, pancreas, small intestine, colon, kidney) (Spence et al., 2011; Mccracken et al., 2014; Takebe et al., 2014; Boj et al., 2015; Morizane et al., 2015; Munera et al., 2017) *via* directed differentiation. However, PSC-derived organoids demonstrate immature, fetal-like, characteristics, which require additional developmental processes to obtain mature organoids. For example, human intestinal organoids (HIOs) derived from PSCs do not possess a full spectrum of markers for mature epithelial cell types, and these HIOs can be matured by surgical implantation into the kidney capsule of immunocompromised non-obese diabetic/*scid*/IL2R γ^null^ (NSG) mice (Watson et al., 2014; Finkbeiner et al., 2015). After 3 months of maturation in mice, human intestinal crypts can be isolated and cultured to derive mouse kidney capsule-matured human intestinal enteroids (kcHIEs).

Alternatively, mouse or human tissue can be used to generate organoids from ASCs that recapitulate organotypic characteristics corresponding to the tissue that was used to derive organoids. For example, mouse intestinal organoids or enteroids can be derived from the leucine-rich repeat-containing G protein-coupled receptor 5 (Lgr5) positive intestinal stem cells. Lgr5+ stem cells isolated from the mouse small intestine can proliferate, differentiate, and organize into 3D enteroids that consist of a single intestinal epithelial cell layer with distinct regions of crypt and villus domains. Analogous to mouse enteroids, ASC-containing patient biopsy-derived human intestinal enteroids (bHIEs) possess a wide spectrum of differentiated cells that reflects the maturity of enteroids derived from ASCs in contrast to fetal-like PSC-derived HIOs. Reviews on PSC- and ASC-derived organoids have been extensively reviewed elsewhere (Rookmaaker et al., 2015; Bartfeld and Clevers, 2017; Schutgens and Clevers, 2020; Lesavage et al., 2022).

Both PSC- and ASC-derived human organoids provide attractive platforms revealing patient- and/or disease subtype-specific differences for disease modeling and drug screening for personalized precision medicine. Broutier et al. (2017) established cancer organoids derived from primary liver cancer (PLC) tissue and demonstrated the heterologous expression of cancer markers depending on PLC subtypes including hepatocellular carcinoma, cholangiocarcinoma and the combined tumor. Despite gene mutations commonly found in tumor tissue, gene set enrichment analysis (GSEA) and next-generation sequencing (NGS) analysis revealed that each subtype of PLC exhibited transcriptomic alterations in gene mutations. Accordingly, they identified candidates of biomarkers for drug screening tests as therapeutic agents for PLC subtypes. Seidlitz et al. (2019) used normal and tumorigenic gastric organoids from patients, and analyzed whole genome sequencing to investigate a broad mutational spectrum from different types of cancer (Seidlitz et al., 2019). The analysis demonstrated significantly high variance in the mutation rate per megabase pair among the patient-derived organoids (PDOs) implying the diversity of pathways and response to treatments. Sachs et al. (2019) established biopsy-derived airway organoid from lung cancer patients and the sequencing analysis determined mutations of *ALK*, *EMR1*, *MDN1*, *STK11*, *TP53*, *KRAS*, and *ERBB2*, which are tumor-related genes (Sachs et al., 2019). Importantly, they also examined differential response to anti-cancer drugs in the airway organoids possessing different mutations, implying that different strategies of treatment are necessary depending on the lung cancer subtypes. Hubert et al. (2016) established the protocol of patient-derived glioblastoma cancer stem cell organoids, and subsequently demonstrated that the alteration of lipid metabolism by upregulation of fatty acid desaturase in glioblastoma organoids, implying that metabolism may vary among different subtypes (Hubert et al., 2016). Additionally, Lee S. H. et al. (2018) used bladder cancer organoids to discover that epigenetic regulators such as *ARID1A*, *KMT2C*, *KMT2D*, and *KDM6A* are mutated as in the bladder cancer tissue, suggesting the differential epigenetics in human cancer (Lee S. H. et al., 2018). They also examined the differential responses to cancer treatment agents among different tumor subtypes from the subjects. Together, these studies using tumorigenic PDOs uncovered fundamental information that could be utilized for *in vitro* drug screening and potential translational applications. However, dysregulation of circadian rhythms, alterations of clock-controlled genes, and potential usages of circadian rhythms for disease treatments have not been thoroughly investigated using organoid models.

Do organoids possess circadian rhythms? We discovered that PSC-derived immature human intestinal organoids (HIOs) do not possess robust circadian rhythms (Rosselot et al., 2021). In contrast, mouse kidney capsule-matured human intestinal enteroids (kcHIEs) show robust circadian rhythms that are comparable to biopsy-derived human intestinal enteroids (bHIEs) based on the expression of a bioluminescence *Bmal1-luciferase* reporter (Rosselot et al., 2021). RNA-Seq analysis of both mouse and human enteroids show that between 3 and 10% of genes are under the control of the intestinal circadian clock (Rosselot et al., 2021). Importantly, we observe appropriate phase relationships between canonical clock genes and eight conserved rhythmic signaling pathways in mouse and human enteroids including circadian clock, cell cycle, and metabolism of carbohydrates (Rosselot et al., 2021). Intriguingly, we observe conserved phases for six signaling pathways including the circadian clock, but two pathways, cell-cell communication and neurotrophin signaling pathways, show dramatically different phases between mouse and human enteroids. Notably, we discovered that *Rac1*, a small GTPase directly inactivated by *Clostridium difficile* toxin B (TcdB), shows anti-phasic gene expression profile between mouse and human enteroids determining anti-phasic necrotic cell death phenotypes against TcdB (Rosselot et al., 2021). The above data demonstrate that PDOs could be ideal *in vitro* model systems investigating disease-specific alterations of circadian rhythms and clock-controlled signaling pathways.

**FIGURE 1:**
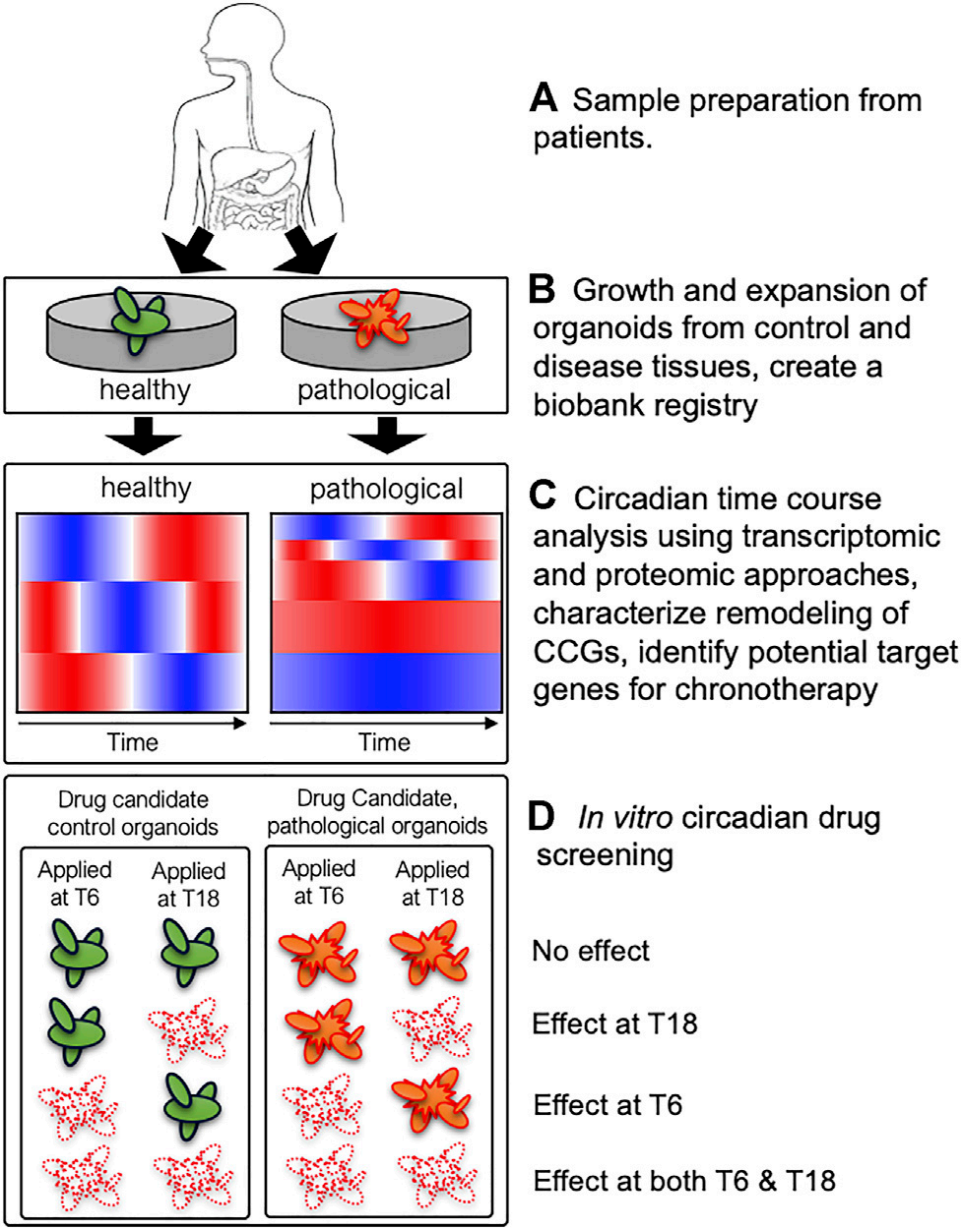
A schematic diagram describing utilization of patient-derived organoids (PDOs) to uncover remodeling of clock-controlled genes (CCGs) and potential circadian drug screening. **(A)** Healthy and pathological tissues (containing stem-cell niche) from biopsies can be used to derive paired control (green) and pathological (orange) organoids from the same patient. **(B)** PDOs are expanded to establish organoid cultures and a subsequent biobank registry containing a library of PDOs from multiple subjects. **(C)** Circadian transcriptomic and proteomic analyses will uncover remodeling of CCGs in control vs. pathological organoids and identify potential target genes for chronotherapy. Cartoons of heatmaps illustrate a potential reduction of rhythmic genes in pathological organoids compared to control organoids. **(D)** Systematic *in vitro* circadian drug screening may reveal different phenotypes between control and pathological organoids with respect to efficacy and circadian timing of drug efficacy, which will provide critical information designing chronotherapeutic regimens. Four potential scenarios (no effect, effect at T6 or T18, and effect at both T6 and T18) are shown for control and pathological organoids. If pathological organoids (orange) show similar cell death (white) at both time points (effects at both T6 and T18) while control organoids (green) showing time-dependent efficacy (effect at T6 or T18) to candidate drugs, then one could design a chronotherapeutic regimen to target pathological organoids while minimizing toxicity to control organoids.

As discussed above, remodeling of clock-controlled genes and signaling pathways has been previously observed in mouse liver in different genetic and external perturbation conditions resulting in different metabolic phenotypes (Hatori et al., 2012; Eckel-Mahan et al., 2013; Zarrinpar et al., 2016; Manella et al., 2021). Hence, we hypothesize that pathological tissues possess altered circadian rhythms and/or clock-controlled genes and signaling pathways exacerbating disease progression. However, it is not feasible to collect human tissues over circadian cycle with fine time resolution (e.g., every 2 h for 48 h). Therefore, paired PDOs derived from control and pathological tissues (e.g., colorectal cancer) from the same patient will provide unique platforms to characterize alterations of clock-controlled genes and signaling pathways using high-throughput transcriptomics and proteomics (Figure 1). This process will uncover remodeling of CCGs in the organoids derived from pathological tissues highlighting altered rhythmicity and/or phase information between control and pathological organoids, which will identify potential circadian target genes for chronotherapeutic interventions. Importantly, this process will also determine whether existing drug target genes show altered temporal patterns between control and pathological organoids. If there exist distinct temporal patterns of existing drug target genes between control and pathological organoids, then this information can be used to design chronotherapeutic regimens maximizing the efficacy of drugs to pathological organoids while minimizing side effects to control organoids. Collected high-throughput time course data from both control and pathological organoids will enable scientists to rationally design potential chronotherapeutic regimens and validate them using PDOs in a disease-specific manner.

## Concluding Remarks

In this minireview, we highlighted the importance of peripheral clocks and potential uses of human organoids to investigate peripheral organ-specific clock functions and remodeling of clock-controlled genes and signaling pathways in pathological tissue-derived organoids using high-throughput transcriptomics and proteomics. It is important to emphasize that mature organoids derived from patients’ tissue biopsy are ideal to generate such data, because the maturity of organoids impacts robustness and function of circadian rhythms. The collected transcriptomic and proteomic data will uncover distinct temporal signatures between control and pathological tissue-derived organoids, which will lead to novel chronotherapeutic regimens in a disease-specific manner. However, it will not be trivial to identify optimal chronotherapeutic design for several drug combinations and numerous target genes. Therefore, it will be necessary to construct mathematical models based on temporal information of key drug target genes and employ computational methods to narrow down testable chronotherapeutic regimens. Rationally designed chronotherapeutic regimens can be validated *in vitro* using PDOs from numerous patients stored in a biobank registry. In particular, utilization PDOs derived from treatment resistant and responsive patients may uncover distinct temporal profiles of CCGs and signaling pathways identifying distinct timing of treatments and/or novel circadian target genes for the treatment resistant group.

It is important to note that the collected transcriptomic and proteomic data using PDOs reflect tissue- and organ-specific CCGs and signaling pathways in the absence of systemic and entrainment cues. Devoid of systemic and entrainment cues in organoid cultures pose critical challenges of organoid usages for potential translational applications of chronotherapy, because patients’ responses to chronotherapy will depend on integrated systemic and peripheral clocks under different entrainment conditions. On the other hand, these challenges present opportunities to further utilize PDOs to determine organ-specific entrainment to different external conditions. For example, microfluidic devices that enable luminal flow could be utilized to test chronotherapeutic regimens using organoids under different nutrient entrainment conditions (Lee K. K. et al., 2018; Sidar et al., 2019). In addition, it would be interesting to test potential bidirectional relationship between the SCN and peripheral clocks by co-culturing mouse SCN slices and organoids. PDOs can be also tested for their light responses for potential light/dark entrainment studies in organ-specific manner, because different Opsins are expressed in different peripheral tissues and organs. For example, Opn3 is expressed in murine skin, adipocytes, and HIEs (Buhr et al., 2019; Nayak et al., 2020; Rosselot et al., 2021). Finally, it is necessary to further validate the identified chronotherapeutic regimens *in vivo*, because it is critical to make sure that the uncovered temporal information in PDOs persist when they are in communication with the rest of the body. As previous research demonstrated that PDOs can be subcutaneously xenografted into NSG mice and measure their growth and toxicity in the NSG mice (Vlachogiannis et al., 2018; Steele et al., 2019), the identified chronotherapeutic regimens may be tested using NSG mice. Synergistic integration of computational models based on *in vitro* and *in vivo* validations using PDOs and mouse models, respectively, will move us forward to potential translational applications of circadian medicine in a disease specific manner.
